# Respiratory Syncytial Virus Induces B Cell Activating Factor (BAFF) in Airway Epithelium: A Potential Avenue for Mucosal Vaccine Development

**DOI:** 10.3390/v17070946

**Published:** 2025-07-04

**Authors:** Wael Alturaiki, Brian Flanagan

**Affiliations:** 1 Department of Medical Laboratory Sciences, College of Applied Medical Sciences, Majmaah University, Majmaah 11952, Saudi Arabia; 2 Department of Women’s and Children’s Health, Institute of Translational Medicine, University of Liverpool, Alder Hey Children’s NHS Foundation Trust Hospital, Eaton Road, Liverpool L12 2AP, UK

**Keywords:** BAFF, RSV, B cells, BEAS-2B, IFN-β

## Abstract

Respiratory syncytial virus (RSV) is a major etiological agent of lower respiratory tract infections, particularly among infants and the elderly. Activation of B cells in the mucosa and the production of specific neutralizing antibodies are essential for protective immunity against pulmonary infection. B-cell activating factor (BAFF) is a critical survival factor for B cells and has been associated with antiviral responses; however, its regulation during RSV infection remains poorly understood. This study examined BAFF regulation in BEAS-2B cells exposed to RSV or IFN-β. The treatments resulted in a progressive increase in gene expression over time, accompanied by higher protein levels. BAFF mRNA peaked at 12 h post-infection and declined by 48 h, coinciding with the release of soluble BAFF protein into the culture supernatant. Pre-treatment with anti-IFN-β antibodies prior to RSV infection reduced both BAFF mRNA and protein levels, indicating that IFN-β plays a regulatory role in BAFF production by airway epithelial cells. Western blot analysis revealed membrane-bound BAFF (~31 kDa) in non-infected cells, with elevated expression at 24 h post-infection. By 48 h, this form was cleaved into a soluble ~17 kDa form, which was detected in the supernatant. Immunostaining further demonstrated reduced surface expression of membrane-bound BAFF in RSV-infected cells compared to uninfected controls, suggesting that RSV infection promotes the cleavage and release of BAFF into the extracellular environment. Additionally, the release of BAFF was not affected by furin convertase inhibition or ER–Golgi transport blockade, indicating a potentially novel cleavage mechanism. Co-culturing BAFF produced by BEAS-2B cells with isolated B cells enhanced B cell viability. Overall, these results indicate that RSV infection stimulates BAFF production in airway epithelial cells through a pathway involving IFN-β, potentially contributing to B cell activation and promoting local antibody-mediated immunity. Understanding this mechanism may offer valuable insights for improving mucosal vaccine strategies and enhancing immunity against respiratory pathogens.

## 1. Introduction

Respiratory syncytial virus (RSV) is a major respiratory pathogen that primarily affects infants and young children, often leading to severe lower respiratory tract infections such as bronchiolitis and pneumonia [1]. Mucosal surfaces serve as the primary entry point for many respiratory pathogens, including RSV [2]. Therefore, understanding the role of mucosal immunity, particularly the involvement of cytokines that enhance B cell activation and antibody production, is essential for developing effective vaccines and therapies targeting RSV and similar respiratory viruses. Mucosal immunization, especially via the respiratory tract, has the potential to induce the production of specific antibodies, including secretory Immunoglobulin A (IgA), which plays a key role in neutralizing pathogens at mucosal surfaces [3]. Compared to systemic immunity, mucosal immunity offers several advantages, most notably its ability to trigger localized immune responses. This localized activation results in faster and more targeted protection while also helping to prevent the spread of infection throughout the body [4,5]. Recent studies have also shown that mucosal vaccination can stimulate both local and systemic immune responses, further supporting its promise as a strategy for developing effective respiratory vaccines [5,6]. For example, vaccines that effectively target mucosal inductive sites can enhance early immune activation, thereby reducing the severity of RSV-related illnesses [7]. A key molecule involved in promoting mucosal immune responses is B-cell Activating Factor (BAFF), which plays an essential role in B cell development, survival, and antibody production [8,9]. Higher BAFF expression has been correlated with enhanced humoral responses post-vaccination, implying that regulating BAFF activity may offer a potential method for strengthening mucosal immunity [10]. Additionally, BAFF has been shown to sustain antibody production over time when administered after vaccination, indicating its potential utility as an adjuvant in mucosal vaccine formulations [8,11]. Therefore, a deeper understanding of the molecular and cellular mechanisms by which BAFF influences mucosal immunity and antibody generation is crucial for optimizing vaccine design against respiratory infections, including RSV [12,13]. This study investigated whether stimulation with IFN-β or infection with RSV promotes BAFF expression in BEAS-2B airway epithelial cells, examined the involvement of IFN-β signaling in this process, and explored the potential mechanism responsible for BAFF proteolytic cleavage following RSV exposure.

## 2. Material and Methods

### 2.1. RSV Challenge and IFN-β Treatment of BEAS-2B Airway Epithelial Cells

BEAS-2B airway epithelial cells (1.5 × 10^5^ cells/well) were seeded in 12-well plates and cultured until approximately 80% confluence was reached. The culture medium (DMEM) was then removed, and cells were washed twice with serum- and antibiotic-free medium. Subsequently, 250 µL of serum- and antibiotic-free medium was added to each well. Subsequently, the cells were exposed to RSV-A2 at different infection levels, using multiplicities of infection (MOI) of 0.025, 0.25, 1, and 2.5 plaque-forming units per cell. Uninfected cells served as negative controls. The plates were gently agitated at 37 °C for 2 h to facilitate infection. Following this, 250 µL of complete DMEM medium was added to each well, and cells were incubated at 37 °C for various time intervals (0, 3, 6, 12, 24, and 48 h). In parallel experiments, BEAS-2B cells were treated with varying concentrations of IFN-β and incubated for the same time intervals. Following incubation, cells were collected through centrifugation, and culture supernatants were separated. Total RNA was isolated using the RNeasy Mini Kit (QIAGEN, Hilden, Germany) following the protocol provided by the supplier. Complementary DNA (cDNA) was generated, and cytokine gene expression was measured by real-time PCR.

### 2.2. Inhibition of IFN-β Follwing RSV Challenge

To assess whether regulation of BAFF in BEAS-2B cells was a direct result of RSV exposure or indirectly driven by infection-induced IFN-β, cells were either treated or left untreated with a neutralizing IFN-β antibody (eBioscience, San Diego, CA, USA). BEAS-2B cells were plated in 96-well plates at a density of 2 × 10^4^ cells/mL and received 5 μL of antibody to reach a final concentration of 20 μg/mL. BAFF transcript levels were measured using RT-PCR at 12 and 48 h following infection. Additionally, supernatants from the cultures were harvested and concentrated using Centriplus YM-10 filters following the supplier’s protocol.

### 2.3. Assessment Survival of B Cells Using MTT Assay

To assess the biological activity of BAFF secreted by epithelial cells, BEAS-2B cells were cultured in complete DMEM and infected with RSV strain A2 at (MOI) of 1 for 48 h. Cell culture supernatants were then concentrated using Centriplus YM-10 centrifugal filters. Human B cells were purified using a negative selection enrichment kit and seeded at 2 × 10^5^ cells per 100 μL in 96-well flat-bottom plates. Cells were cultured in RPMI 1640 medium supplemented with 10% fetal bovine serum, 100 U/mL penicillin, and 100 μg/mL streptomycin. The experimental groups included media control, B cells alone, B cells treated with 10 ng/mL recombinant human BAFF (R&D Systems, Minneapolis, MN, USA), and B cells exposed to a 1:20 dilution of supernatant from RSV-infected BEAS-2B cultures. Cells were incubated for five days. To assess cell viability, 20 μL of MTT solution was added during the final 4 h, followed by 100 μL of detergent reagent. Plates were mixed thoroughly, and absorbance was recorded at 540 nm using a plate reader. All experiments were performed in triplicate, and mean values were reported.

### 2.4. Identification of BAFF Forms in BEAS-2B Cells Post-RSV Challenge

To investigate the different forms of BAFF produced after RSV infection, BEAS-2B cells were seeded in 24-well plates at a concentration of 2.5 × 10^6^ cells/mL and exposed to RSV at an MOI of 1, followed by incubation for up to 24 h. Afterward, cells were harvested using a scraper, rinsed with PBS, and lysed in SDS buffer supplemented with 10% DTT and protease inhibitors. Samples were heated at 100 °C for 3 min, and 10 µL from each was loaded onto a 12% SDS-PAGE gel. Protein separation was carried out at 140 V for one hour and then transferred to nitrocellulose membranes using the Trans-Blot Turbo device (Bio-Rad, Hercules, CA, USA) for 7 min. Membranes were blocked with 5% skim milk in TBST for 30 min, followed by overnight incubation with a BAFF-specific primary antibody at 4 °C. After washing, membranes were treated with an HRP-linked secondary antibody for one hour at room temperature. Protein bands were visualized using enhanced chemiluminescence (ECL) and captured on X-ray film.

### 2.5. Investigation of How RSV Challenge in BEAS-2B Cells Promotes BAFF Cleavage

To determine whether BAFF processing involves furin-like enzymes, BEAS-2B airway epithelial cells were infected with RSV at an MOI of 1. Six hours later, cells were exposed to chloromethylketone (a furin inhibitor; Fisher Scientific, Hampton, NH, USA) at concentrations of 50 or 100 μM and incubated for an additional 24 or 48 h. To further investigate the cleavage mechanism, additional sets of RSV-infected or IFN-β–treated cells received Brefeldin A at the 6 h mark and were maintained under similar conditions. Cells were collected via scraping, rinsed in PBS, and centrifuged at 5000 rpm for 10 min. Pelleted cells were lysed in SDS buffer containing 10% DTT and a protease inhibitor cocktail, and proteins were evaluated by Western blot analysis.

### 2.6. BAFF Protein Quantification and Immunofluorescence Staining

Soluble BAFF protein was quantified in the supernatants of BEAS-2B cells following RSV infection or IFN-β treatment using an ELISA kit (R&D Systems, Minneapolis, MN, USA; Cat# DBLYS0B), following the supplier’s guidelines. Protein concentrations were determined using KC Junior software (BioTek, Winooski, VT, USA). To further assess BAFF expression and localization, immunofluorescence staining was performed. BEAS-2B cells were seeded onto glass coverslips placed in 12-well culture plates at a density of 5 × 10^4^ cells per well, followed by RSV infection. After 48 h, cells were fixed with cold methanol for 10 min, washed, and permeabilized with 1% Tween-20 (two 5 min washes). Blocking was performed using 3% BSA for 30 min. Cells were then incubated for 1 h in the dark at room temperature with directly labeled antibodies: anti-BAFF-FITC (Abcam, Cambridge, UK) and anti-RSV (Abcam, Cambridge, UK). Afterward, cells were washed twice in 1% Tween-20 (5 min each) and treated with a secondary antibody against RSV (R&D Systems, Minneapolis, MN, USA) diluted in blocking buffer for 1 h at room temperature. Additional washes were performed using PBS supplemented with 1% Tween-20 (5 min each). Nuclear staining was carried out using DAPI (Sigma, Kawasaki City, Japan), diluted in PBS and applied for 3 min. Coverslips were subsequently rinsed in PBS with Tween-20 for 5 min, mounted with a drop of mounting medium (R&D Systems, Minneapolis, MN, USA), and sealed using a PAP pen. Imaging was conducted via confocal microscopy (LEICA DM2500, Leica Microsystems, Wetzlar, Germany) using appropriate fluorescence filter sets. BAFF, RSV, and nuclear signals were visualized in green, red, and blue channels, respectively.

### 2.7. Statistical Analysis

Data are presented as mean ± standard error of the mean (SEM). Statistical analysis was performed using one-way ANOVA followed by Bonferroni’s post hoc test, conducted in GraphPad Prism version 5.0. Statistical significance was defined as follows: *p* < 0.05 (*), *p* < 0.001 (**), and *p* < 0.0001 (***), representing increasing levels of confidence.

## 3. Results

### 3.1. RSV Infection Enhances BAFF Gene and Protein Expression in BEAS-2B Cells in a Time- and Dose-Dependent Manner

**Figure 1:**
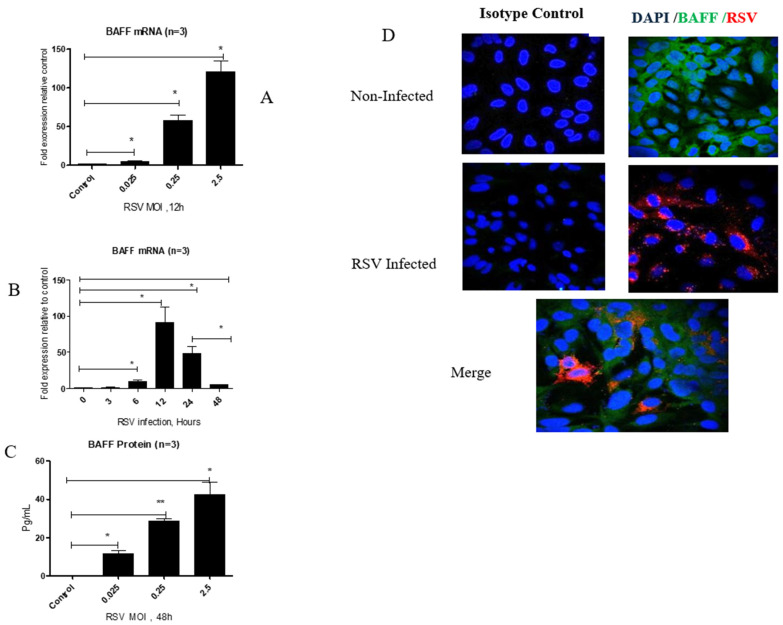
BAFF gene and protein levels in BEAS-2B ells post-RSV challenge (**A**) RT-PCR quantification of BAFF gene expression in BEAS-2B cells infected with RSV at 0, 3, 6, 12, 24, and 48 h post-infection. (**B**) BAFF gene expression measured at 12 h post-infection in cells infected at MOIs of 0.025, 0.25, and 2.5. (**C**) ELISA measurement of BAFF protein concentrations in cell culture supernatants at 48 h post-infection at different MOIs. (**D**) Immunofluorescence imaging of BAFF (green) and RSV (red) in infected BEAS-2B cells at 12 h post-infection compared with non-infected controls. Nuclei appear in blue. Isotype controls were included to verify staining specificity. Data shown are averages from three independent experiments (n = 3). Images were obtained using confocal microscopy at 40× magnification. Statistical significance was defined as follows: *p* < 0.05 (*) and *p* < 0.001 (**), representing increasing levels of confidence.

BAFF gene expression was analyzed in BEAS-2B cells infected with RSV at various intervals (0, 3, 6, 12, 24, and 48 h). The levels significantly increased at 6 h, reached their highest at 12 h, and subsequently decreased at 24 and 48 h post-infection (Figure 1A). To investigate the impact of viral concentration, BAFF gene expression was measured 12 h after infecting cells with RSV at multiple multiplicities of infection (MOIs: 0.025, 0.25, and 2.5). BAFF expression notably increased with higher virus concentrations, with maximum levels observed at MOI 2.5 (Figure 1B). Corresponding with gene expression, BAFF protein in cell culture supernatants also significantly increased at 48 h in a dose-responsive pattern, showing peak secretion at MOI 2.5 (Figure 1C). Additionally, immunofluorescence staining performed 12 h post-infection demonstrated cytoplasmic distribution of BAFF within RSV-infected BEAS-2B cells (Figure 1D). Notably, RSV-infected cells exhibited lower surface membrane-bound BAFF expression than uninfected controls, accompanied by enhanced release of soluble BAFF.

### 3.2. IFN-β Treatment Induces BAFF Gene and Protein Expression in BEAS-2B Cells

**Figure 2:**
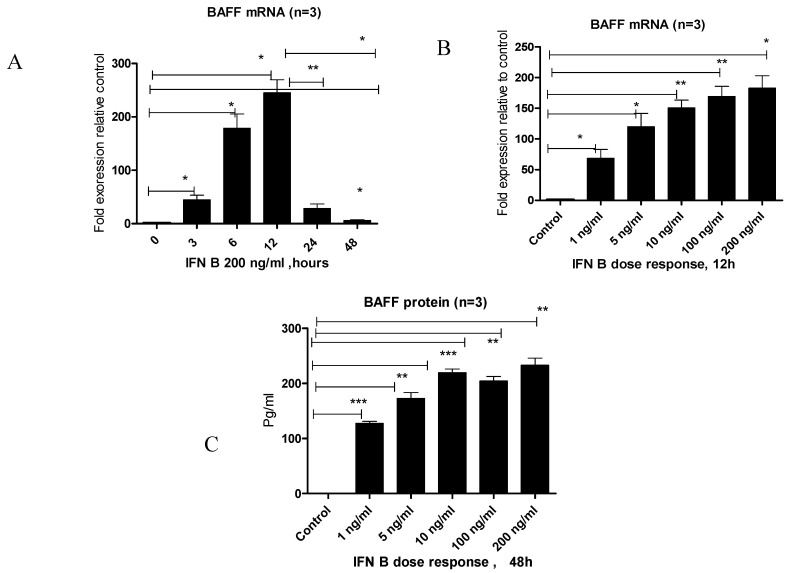
IFN-β induces BAFF gene and protein levels in BEAS-2B cells. (**A**) RT-PCR measurement of BAFF gene levels in BEAS-2B cells at different time intervals (0, 3, 6, 12, 24, and 48 h) after IFN-β treatment. (**B**) BAFF gene expression quantified at 12 h post-stimulation with various IFN-β concentrations. (**C**) ELISA determination of BAFF protein concentrations in culture supernatants 48 h post-stimulation at different IFN-β doses. Data represent the mean ± SD from three independent experiments (n = 3). Statistical significance was defined as follows: *p* < 0.05 (*), *p* < 0.001 (**), and *p* < 0.0001 (***), representing increasing levels of confidence.

To investigate the effect of IFN-β on BAFF expression, BEAS-2B cells were stimulated with IFN-β, and BAFF gene and protein levels were assessed at multiple time points and concentrations. BAFF gene levels increased significantly at 3 and 6 h, reached a peak at 12 h post-stimulation, and declined thereafter at 24 and 48 h (Figure 2A). Subsequently, stimulation with increasing concentrations of IFN-β led to a marked elevation in BAFF gene expression at 12 h, with the highest expression observed at the highest concentration tested (Figure 2B). Consistently, BAFF protein levels in the culture supernatant at 48 h post-treatment also significantly increased with higher IFN-β doses (Figure 2C).

### 3.3. Does RSV Infection Induce BAFF Through an IFN-β-Mediated Mechanism?

**Figure 3:**
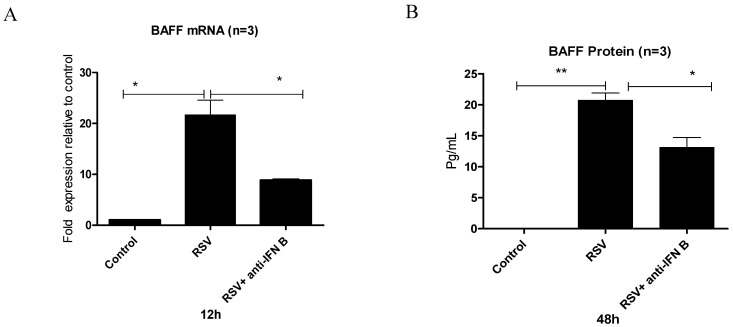
IFN-β is required for BAFF expression in BEAS-2B cells infected with RSV. BEAS-2B cells were infected with RSV at MOI of 1 and either pretreated or not with a neutralizing antibody against IFN-β. (**A**) BAFF gene levels were analyzed by RT-PCR 12 h after infection and are shown as fold change relative to untreated controls. (**B**) At 48 h, BAFF protein concentrations in the culture medium were quantified by ELISA and reported as pg/mL. Blocking IFN-β activity prior to infection led to a clear reduction in both mRNA and protein levels compared to RSV-only treatment. Results represent the average of three separate experiments (n = 3). Statistical significance was defined as follows: *p* < 0.05 (*) and *p* < 0.001 (**), representing increasing levels of confidence.

To assess whether BAFF upregulation following RSV challenge is directly mediated by the virus or indirectly regulated through IFN-β signaling, BEAS-2B cells were challenged with RSV with or without a neutralizing antibody against IFN-β. A marked increase in BAFF transcripts was observed at 12 h, and elevated protein levels were detected at 48 h post-infection relative to untreated cells. However, when cells were pretreated with the neutralizing antibody before RSV exposure, both BAFF gene and protein production were significantly reduced (Figure 3A,B), indicating that IFN-β contributes substantially to BAFF upregulation during RSV infection.

### 3.4. Epithelial Cell-Derived BAFF Following RSV Infection Enhances B Cell Survival

**Figure 4:**
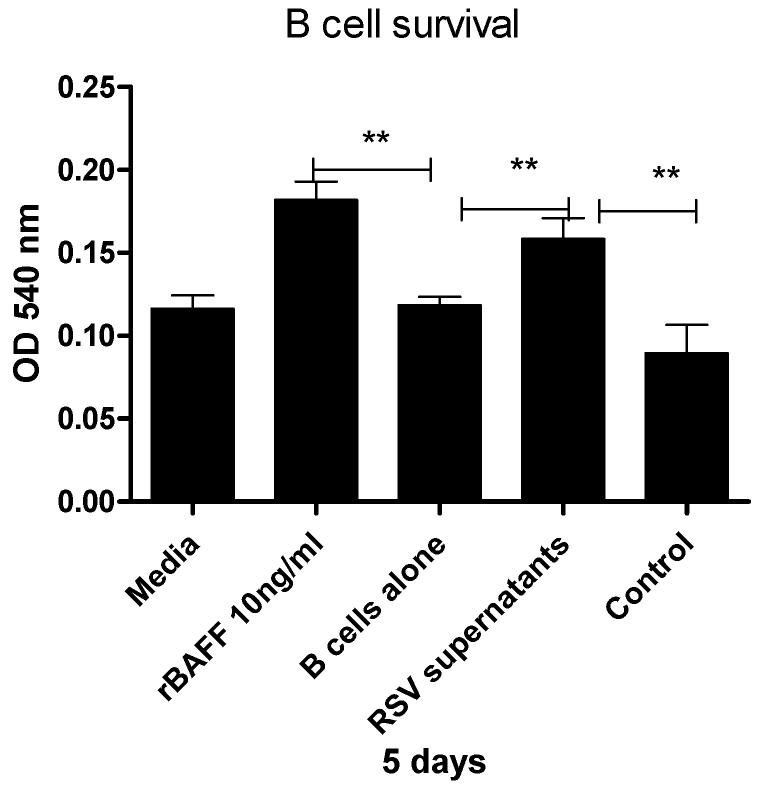
BAFF from RSV-challenged BEAS-2B cells enhances B cell survival. Purified human B cells were cultured for 5 days under one of the following conditions: medium alone, 10 ng/mL recombinant BAFF (rBAFF), or concentrated supernatants collected at 48 h from BEAS-2B cells, either challenged with RSV or left untreated. B cell viability was evaluated using the MTT assay, with optical density (OD) measured at 540 nm. Results represent the average of three independent experiments. Statistical significance was defined as follows: *p* < 0.001 (**), representing increasing levels of confidence.

To determine whether BAFF released by RSV-challenged epithelial cells contributes to B cell survival, purified human B cells were cultured for 5 days with or without concentrated supernatants collected at 48 h from BEAS-2B cells challenged with RSV or left untreated. Cell viability was measured using the MTT assay. As expected, recombinant human BAFF significantly enhanced B cell survival relative to media alone. Moreover, supernatants obtained from RSV-challenged cultures supported B cell viability to a greater extent than those from unchallenged controls, which showed reduced survival (Figure 4).

### 3.5. BAFF Isoform Expression in BEAS-2B Cells Post-RSV Challenge

**Figure 5:**
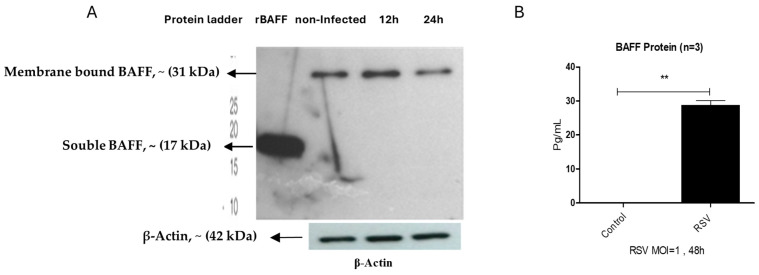
Evaluation of BAFF forms in BEAS-2B cells after RSV exposure. (**A**) BEAS-2B cells were exposed to RSV (MOI = 1) and collected at 12 and 24 h post-infection. Protein extracts were analyzed by Western blot under reducing conditions. The membrane-bound form of BAFF (~31 kDa) was present in both control and infected samples. A molecular weight marker was included for size reference, and β-actin served as the internal loading control (42 kDa). Recombinant human BAFF (~17 kDa) served as a reference for the soluble form. (**B**) Soluble BAFF levels in culture supernatants were quantified at 48 h post-challenge using a human-specific BAFF ELISA. Statistical significance was defined as follows: *p* < 0.001 (**), representing increasing levels of confidence.

Western blot analysis demonstrated that uninfected BEAS-2B cells predominantly expressed membrane-associated BAFF (~31 kDa). Upon RSV challenge, this form remained present at 12 h but showed a noticeable decline by 24 h (Figure 5A). At 48 h, ELISA measurements revealed a substantial elevation of the soluble BAFF isoform (~17 kDa) in the supernatants of infected cultures compared to uninfected controls (Figure 5B), suggesting that RSV promotes the cleavage and secretion of soluble BAFF over time.

### 3.6. Characterization of BAFF Cleavage Pathways

**Figure 6:**
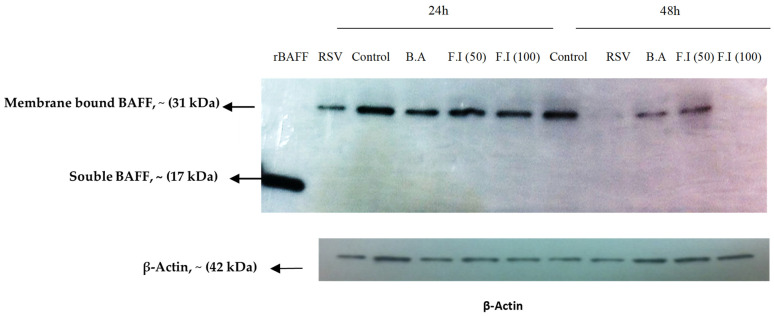
Detection of BAFF expression in BEAS-2B cells treated with Brefeldin A or furin inhibitor after RSV exposure. BEAS-2B cells were exposed to RSV (MOI = 1) and subsequently treated with either Brefeldin A or a furin convertase inhibitor at concentrations of 50 μM and 100 μM. Cells were collected at 24 and 48 h following infection and examined by Western blot. The membrane-bound form of BAFF (~31 kDa) was observed in uninfected cells and remained detectable at 24 h post-infection, though its levels declined by 48 h. β-actin served as the internal control (42 kDa), and recombinant BAFF (rBAFF) was used as a positive control for the soluble isoform (~17 kDa).

To explore whether RSV-induced production of soluble BAFF involves cleavage by furin-like enzymes, BEAS-2B cells were infected with RSV and exposed to a furin convertase inhibitor. At 24 and 48 h post-infection, membrane-bound BAFF was evident in uninfected cells but was reduced following RSV exposure, particularly by 48 h. Inhibition of furin activity did not restore membrane BAFF; in fact, treatment with the higher inhibitor dose (100 μM) led to a further decline in its expression. Moreover, treatment with brefeldin A had no detectable impact on membrane BAFF, suggesting that soluble BAFF is likely released through cleavage at the plasma membrane rather than by conventional secretion through the ER–Golgi pathway (Figure 6).

## 4. Discussion

This study shows that RSV infection, along with type I interferon signaling, specifically IFN-β, enhances BAFF expression in human airway epithelial cells (BEAS-2B). In the absence of infection, BAFF was present at low levels, mainly in its membrane-bound form. After RSV exposure, BAFF mRNA increased at 3 and 6 h, reached its highest level at 12 h, and declined by 48 h (Figure 1A). At 12 h post-infection, BAFF mRNA levels were also higher with increasing viral dose, with the strongest expression observed at MOI 2.5 (Figure 1B). BAFF is initially produced on the cell membrane and can be processed into a soluble form through cleavage by furin enzymes [14]. This is supported by the observation that membrane-bound BAFF decreased over time, while the soluble form became more detectable in the culture medium by 48 h (Figure 1C). Consistent with this, immunofluorescence staining showed reduced surface BAFF in infected cells, whereas uninfected cells maintained stronger membrane-associated signals (Figure 1D). Collectively, these results indicate that RSV challenge facilitates the shedding of soluble BAFF from the cell surface through proteolytic cleavage.

To assess the role of interferon signaling in regulating BAFF expression, BEAS-2B cells were treated with IFN-β. A clear increase in BAFF mRNA was detected, which progressed over time and with higher IFN-β concentrations, reaching peak levels at 12 h (Figure 2A). Both gene and protein expression were strongly enhanced at elevated IFN-β doses, suggesting that IFN-β functions as an upstream regulator of BAFF (Figure 2B,C). Interestingly, even low IFN-β concentrations (1 ng/mL) were sufficient to trigger a notable increase in BAFF expression. To assess whether this pathway is involved during RSV infection, IFN-β signaling was blocked prior to infection. Inhibition led to a substantial decrease in BAFF mRNA and protein, supporting a key role for IFN-β in the induction of BAFF during RSV exposure (Figure 3A,B). These observations are consistent with earlier studies proposing that IFN-β is critical for initiating BAFF expression in epithelial cells [15,16].

To evaluate the functional activity of BAFF released by RSV-challenged airway epithelial cells, we examined whether supernatants from BEAS-2B cells challenged with RSV could enhance the survival of primary human B cells. As a positive control, recombinant human BAFF significantly increased B cell viability, confirming the responsiveness of the assay. Notably, concentrated supernatants from RSV-challenged cultures significantly enhanced B cell survival compared to those from unchallenged cells, which showed minimal or even negative effects on viability. These findings suggest that RSV challenge induces the release of biologically active soluble BAFF capable of supporting B cell maintenance in vitro (Figure 4). This observation is consistent with previous findings by Kato et al. [16], who showed that BAFF released from BEAS-2B cells in response to poly I:C also promoted B cell survival through a BAFF-dependent mechanism. Although we did not evaluate the effect of BAFF-specific inhibitors in this study, the increase in B cell viability observed following RSV exposure supports the functional relevance of the soluble BAFF detected in our assays. In addition, influenza infection has been shown to dramatically upregulate BAFF expression in bronchial epithelial cells, indicating that these cells actively contribute to local immune responses [17,18].

Moreover, BAFF derived from epithelial cells has also been found to promote B cell activation and plasma cell differentiation, leading to enhanced antibody production [19]. This process is critical for maintaining robust antibody levels at mucosal surfaces, where initial host–pathogen interactions typically occur. Furthermore, exogenous administration of BAFF has been shown to prevent the decline of antibody titers following immunization, as demonstrated in models using heat-killed *Pseudomonas aeruginosa* [8]. These findings suggest that modulating BAFF levels may serve as an effective strategy for sustaining long-term antibody responses, particularly against respiratory pathogens [15]. In addition, experimental evidence indicates that BAFF plays a role in the development of memory B cells, which are essential for long-term immunity. In the context of severe RSV infection, BAFF produced by infected airway epithelial cells promotes local antibody production [15,20], a process mediated through TLR pathway activation [21]. Notably, the immunomodulatory functions of BAFF extend beyond B cells, as it also enhances T cell responses and intercellular immune communication. Elevated BAFF levels have been associated with improved Th1-mediated immunity and increased production of virus-specific neutralizing antibodies [22,23]. Collectively, these findings underscore the pivotal role of BAFF in orchestrating both humoral and cellular immune responses during respiratory viral infections, and highlight its potential as a promising target for vaccine development and immunotherapeutic interventions.

Western blot analysis of unstimulated epithelial cells revealed the presence of membrane-associated BAFF (~31 kDa), consistent with previous findings [16]. Following RSV infection, mBAFF expression was detectable as early as 12 h but began to decline slightly by 24 h (Figure 5A), suggesting that BAFF may undergo post-translational processing or downregulation as infection progresses. At 48 h post-infection, soluble BAFF (sBAFF; ~17 kDa) levels in the culture supernatant were significantly increased, as measured by ELISA (Figure 5B). These results are in line with observations by Kato et al., who showed that poly I:C (a TLR3 ligand) stimulates BAFF transcription and cleavage in airway epithelial cells via an IFN-β-dependent autocrine mechanism. However, our findings suggest a temporally regulated BAFF response specific to RSV infection, characterized by early surface expression of mBAFF followed by the generation of sBAFF. The observed decrease in mBAFF concurrent with the rise in sBAFF supports the hypothesis that membrane-bound BAFF is cleaved and released extracellularly. Notably, the mechanism underlying this cleavage during RSV infection may differ from previously reported furin-dependent pathways. Specifically, treatment with a furin inhibitor following RSV infection did not lead to an accumulation of mBAFF, nor did it significantly reduce sBAFF levels. In contrast, higher concentrations of the inhibitor (100 µM) resulted in a further decrease in mBAFF, potentially indicating cytotoxic or off-target regulatory effects (Figure 6). To further explore the site of BAFF processing, BEAS-2B cells were treated with Brefeldin A (BA), a compound that blocks protein transport from the endoplasmic reticulum to the Golgi apparatus. BA treatment did not alter membrane BAFF expression, supporting the hypothesis that sBAFF is cleaved at the cell surface rather than secreted through intracellular vesicular trafficking. Together, these findings point to a distinct post-translational regulatory mechanism of BAFF in RSV-infected airway epithelial cells, which appears to be independent of the canonical furin-dependent cleavage observed in poly I:C-stimulated cells. While dsRNA activates type I IFN signaling and furin-mediated cleavage via the JAK/STAT pathway, RSV may utilize a virus-specific mechanism to modulate epithelial immune responses, underscoring the complexity and specificity of host–pathogen interactions during viral infections.

## 5. Conclusions and Future Remarks

RSV exposure and IFN-β treatment both led to a marked increase in BAFF expression at the mRNA and protein levels in BEAS-2B cells. Gene expression peaked at 12 h and declined by 48 h, coinciding with the appearance of sBAFF in the culture medium. This response was dependent on IFN-β, indicating its role as an early signal driving BAFF production in airway epithelial cells. Western blot analysis confirmed the presence of the membrane-bound form (~31 kDa) in non-infected cells, while a smaller soluble form (~17 kDa) appeared following infection, likely reflecting cleavage of the membrane-anchored protein. Notably, blocking furin activity did not prevent this cleavage, and brefeldin A treatment had no impact, suggesting that an alternative mechanism is responsible for releasing soluble BAFF from the cell surface. Together, these findings emphasize the contribution of epithelial cells to B cell support via BAFF production. Given BAFF’s role in promoting B cell survival and antibody formation, it may influence mucosal immune responses in the respiratory tract. Further investigation is needed to clarify how BAFF modulates B cell function in this setting and whether it can be used to improve mucosal immune responses in the development of respiratory vaccines.

## Data Availability

Further inquiries can be directed to the corresponding authors.
